# Volumetric Measurement of Subretinal Blebs Using Microscope-Integrated Optical Coherence Tomography

**DOI:** 10.1167/tvst.7.2.19

**Published:** 2018-04-05

**Authors:** S. Tammy Hsu, Hesham Gabr, Christian Viehland, Karim Sleiman, Hoan T. Ngo, Oscar M. Carrasco-Zevallos, Lejla Vajzovic, Ryan P. McNabb, Sandra S. Stinnett, Joseph A. Izatt, Anthony N. Kuo, Cynthia A. Toth

**Affiliations:** 1 Department of Ophthalmology, Duke University School of Medicine, Durham, NC, USA; 2 Department of Ophthalmology, Ain-Shams University, Cairo, Egypt; 3 Department of Biomedical Engineering, Duke University, Durham, NC, USA

**Keywords:** subretinal space, gene therapy, retina, drug delivery, optical coherence tomography

## Abstract

**Purpose:**

We advance studies of subretinal treatments by developing a microscope-integrated optical coherence tomography (MIOCT) image-based method for measuring the volume of therapeutics delivered into the subretinal space.

**Methods:**

A MIOCT image-based volume measurement method was developed and assessed for accuracy and reproducibility by imaging an object of known size in model eyes. This method then was applied to subretinal blebs created by injection of diluted triamcinolone. Bleb volumes obtained from MIOCT were compared to the intended injection volume and the surgeon's estimation of leakage.

**Results:**

Validation of the image-based volume measurement method showed accuracy to ±1.0 μL (6.0% of measured volume) with no statistically significant variation under different imaging settings. When this method was applied to subretinal blebs, four of 11 blebs without surgeon-observed leakage yielded a mean volume of 32 ± 12.5 μL, in contrast to the intended 50 μL volume injected from the delivery device. This constituted a mean difference of −18 μL (mean percent error, 36 ± 25%). For all 11 blebs, the surgeon's estimations of leakage were significantly different from and showed no correlation with the volume loss based on image-based volume measurements (*P* < 0.001, paired *t*-test; intraclass correlation = 0).

**Conclusions:**

We validated an accurate and reproducible method for measuring subretinal volumes using MIOCT. Use of this method revealed that the intended volume might not be delivered into the subretinal space. MIOCT can allow for accurate assessment of subretinal dose delivered, which may have therapeutic implications in evaluating the efficacy and toxicity of subretinal therapies.

**Translational Relevance:**

Use of MIOCT can provide feedback on the accuracy of subretinal injection volumes delivered.

## Introduction

Subretinal injections of therapeutics currently are being investigated as potential treatments for major causes of blindness. Injection of healthy retinal pigment epithelium (RPE) cells into the subretinal space of eyes with age-related macular degeneration (AMD) and Stargardt's macular dystrophy is being studied as a therapy to prevent or reverse photoreceptor injury, as evidence suggests that dysfunction and loss of the RPE leads to photoreceptor damage and vision loss in these diseases.^1–4^ More recently, clinical trials involving subretinal injections of viral vectors as gene therapy for retinitis pigmentosa,^5^ Leber's congenital amaurosis.^6,7^ and Leber's hereditary optic neuropathy^8^ have been conducted. Subretinal injections of recombinant tPA also have been used to manage submacular or subretinal hemorrhage.^9,10^ However, subretinal injections carry risk of complications, including retinal detachment, choroidal hemorrhages, rupture of Bruch's membrane, and reflux of RPE cells or other therapeutics from the needle.^11^ This may lead to insufficient delivery of stem cells into the subretinal space or leakage of viral vectors into the vitreous body, and increase the risk of postoperative vitritis.

Currently, a surgeon's success in accurately placing and estimating the volume of therapeutics delivered beneath the retina is based on surgical experience and en face visualization via the surgical microscope. The accepted practice for determining the volume of a subretinal bleb is by injection of a predetermined volume of the target therapeutic from a calibrated syringe into the subretinal space. If the surgeon does not observe any leakage, it is assumed that all of the injected volume is delivered successfully into the subretinal space.^2,5–8,12,13^ With the rapidly increasing interest in subretinal therapies and progress in clinical trials, there is a need to test this assumption and directly and accurately measure the volumes of therapeutics.

To address this need, we proposed a microscope-integrated optical coherence tomography (MIOCT) image-based method to quantify the volume of therapeutics delivered into the subretinal space. The potential for improved visualization of volumes of therapeutics delivered into the subretinal space has been attempted early in the era of imaging, including with ultrasound,^14^ and shown in previous studies using spectral domain OCT (SD-OCT) to image areas of subretinal injections immediately before and after injection, and three-dimensional (3D) volumes have been postprocessed and reconstructed from the collected data.^15–17^ More recently, live spectral domain microscope-integrated OCT (SD-MIOCT) showing two-dimensional (2D) B-scans have been used intraoperatively to aid in delivery of viral vectors and RPE cells into the subretinal space.^18,19^ Gregori et al.^20^ demonstrated the benefits of SD-MIOCT in subretinal gene therapy delivery; the ultimate goal, he stated, would be to calculate the volume of vector injected. Current generation commercial retinal SD-MIOCT systems, however, have limitations, such as in speed and depth of imaging, that make intraoperative imaging and quantitative assessment of subretinal fluid volumes challenging.^15^

To aid in the quantification of subretinal bleb volumes and address some of the limitations mentioned in previous SD-MIOCT studies, we used a swept-source MIOCT system. Compared to current retinal SD-OCT systems, swept-source OCT has increased depth of imaging (7.4 mm) and is more than three times faster to allow dynamic localization of instruments and complete capture of all subretinal bleb boundaries.^21,22^ In previous swept-source MIOCT studies, live volumetric images of subretinal blebs were captured (Vajzovic LM, et al. *IOVS*. 2017;58:ARVO E-Abstract 3122), thus prompting the question of whether we also could use these 3D images to quantify the volume of subretinal blebs. Accurate and reproducible quantification of subretinal volumes using SD-OCT images has been shown previously for measurement of drusen volumes in AMD,^23^ and, thus, we hypothesize that swept-source MIOCT also can be used to image and measure volumes of subretinal blebs accurately and reproducibly within 10% of the actual volume. Additionally, we hypothesized that swept-source MIOCT image-based volume measurements will be comparable to the current standard, where the volume of the subretinal bleb is equal to the volume injected from a calibrated delivery device under the assumption of that no leakage is observed by the surgeon.

## Methods

To test our hypothesis, we developed and assessed a method for measuring volumes of subretinal blebs in model porcine eyes using images acquired with a swept-source MIOCT prototype system developed at Duke University.^21^ The system uses a 100 kHz Axsun swept-source laser centered at 1060 nm, has a peak sensitivity of 99 dB, allows for up to 7.4 mm depth imaging range, and has an axial resolution in air of 7.8 μm. An electrically-tunable lens allowed for dynamic focus adjustment ensuring that the OCT system was parfocal with the surgical microscope.^24^ We acquired volumetric images with 1000 A-scans/B-scan and 128 B-scans/volume through a contact lens. Lateral dimensions of volumes consisted nominally of either 6 × 6 or 10 × 10 mm. Additionally, volumes were captured at 0° and 90° scan angles to establish intrasession reproducibility.

### Validation of MIOCT Image-Based Volume Measurements

**Figure 1:**
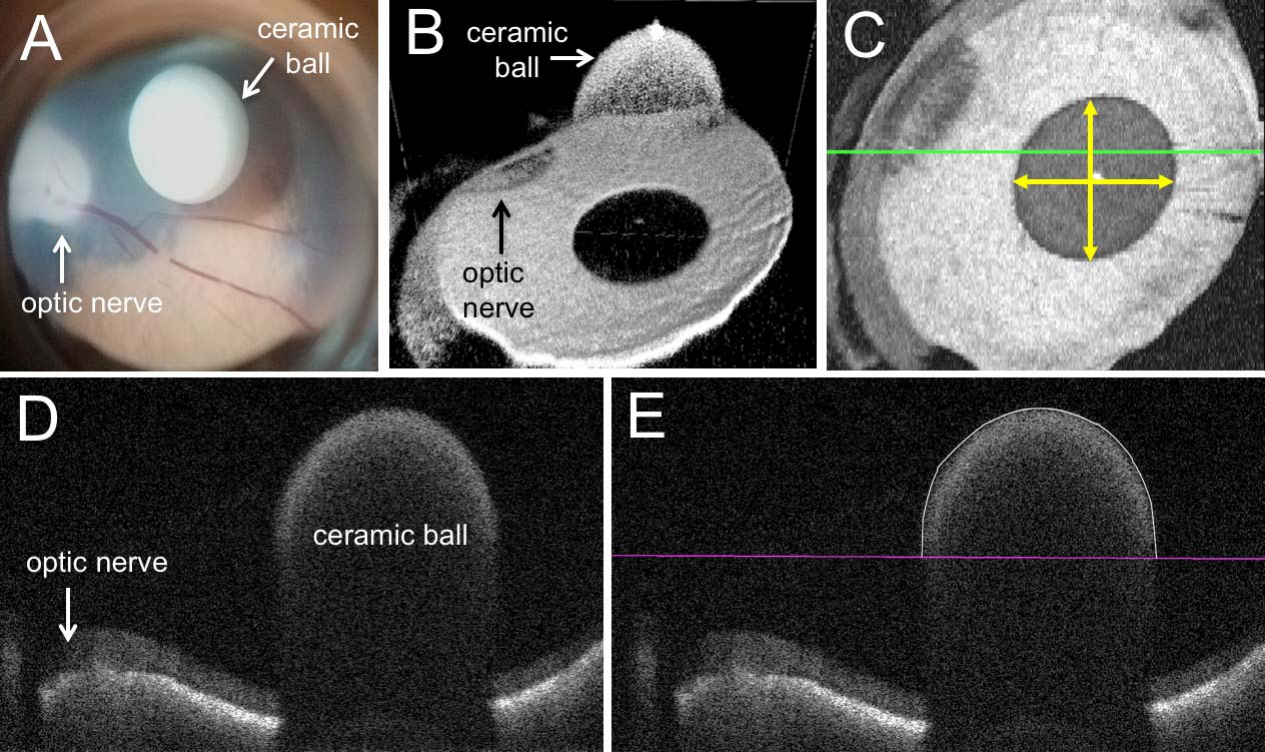
A ceramic ball resting on the retina of a vitrectomized porcine eye: (A) microscope view, MIOCT images in (B) 3D and (C) en face with a *green line* showing (D) a representative corresponding B-scan and (E) segmented area. The *yellow arrows* in the en face image (C) show where the ball diameter was measured in the *X* and *Y* lateral dimensions.

We first calculated volumes from MIOCT images, and assessed the variances due to manual segmentation, different porcine eyes, and different imaging system settings (scan length, scan angle, and focal length). To assess the accuracy of MIOCT image-based volume measurements, we repeatedly imaged a white nonporous alumina ceramic ball (McMaster-Carr, Douglasville, GA). The reported diameter from the manufacturer was 1/8 inches or 3.175 mm (with sphericity equal to 99.9975%). We verified the diameter using electronic calipers (3.17 mm) resulting in a calculated volume of 16.7 μL for comparison with subretinal injection volumes (measured in μL). We placed the ball in a vitrectomized porcine eye through a sclerotomy so that the ball rested on the retina, simulating the approximate space that would be occupied by a bleb formed from a subretinal injection (Fig. 1A). Images of the ball and porcine retina were captured through a flat contact lens on top of the cornea using the MIOCT imaging system settings mentioned above (Fig. 1B).

To calculate the MIOCT image-based volume of the ball, we first manually segmented the volumetric image by tracing the boundaries in the B-scans using DOCTRAP software.^25^ To assess the accuracy and repeatability of manual segmentation of B-scans, a grader repeatedly traced the top curve of the ceramic ball in the same set of B-scans from a single volumetric image. However, unlike the B-scans of a subretinal bleb in which the upper and lower boundaries were clear and easy to segment, the lower half of the ceramic ball was not visible due to shadowing; therefore, to avoid introducing irrelevant error to our method for measuring subretinal bleb volumes, we set the same line in all B-scans of the ball to serve as the lower boundary of the segmentations, producing segmented areas of semicircles throughout the B-scans (Figs. 1D, 1E). The areas of the semicircles then were doubled to convert the areas to those of full circles. The variance in manual segmentation was factored into the propagation of uncertainty^26^ calculations to obtain an overall measure of uncertainty associated with the MIOCT image-based method for measuring volumes.

To convert the total segmented area within all B-scans acquired across the ball from pixels (rectangles on a two-dimensional grid) into units of cubic millimeters (and consequently microliters), we multiplied the number of pixels by the *X*, *Y*, and *Z* dimensions of a voxel (a cuboid on a three-dimensional grid):

\[{\rm{Voxel\ volume}} = X{\rm{\ dimension}} \times Y{\rm{\ dimension}} \times Z{\rm{\ dimension}}\]

\[{\rm{Total\ volume}} = {\rm{voxel\ volume}} \times {\rm{total\ number\ of\ pixels\ from\ all\ segmented\ B}} -\ \rm scans\]

To determine the *X* and *Y* lateral dimensions, we measured the diameter of the ball on the en face MIOCT images in pixels, calibrated that to the known diameter of the ball in millimeters, and calculated the conversion from pixels to millimeters (Fig. 1C). To assess the variation in the *X* and *Y* dimensions of MIOCT images due to differences between porcine eyes, we then repeated these measures under the same imaging system settings in different eyes. The variance in the calibrated *X* and *Y* lateral dimensions were factored into the overall measure of uncertainty associated with the MIOCT image-based method for measuring volumes. The *Z* pitch was calculated from a formula using the number of spectral samples and index of refraction of balanced salt solution (BSS). The accuracy in the Z pitch is an inherent property of the swept source laser clock and the digitizer, and is negligible compared to segmentation error.^27^

We assessed for imaging reproducibility using multiple imaging system settings: (1) lateral scan dimensions (nominally 6 × 6 vs. 10 × 10 mm), (2) scan angle (0° vs. 90°), and (3) focal length of the electrically-tunable lens^24^ (within a visually optimal range ± 1.8 mm from the ideal focus while maintaining parfocality with the surgical microscope). Diameters in the *X* and *Y* lateral dimensions were measured 10 times for each en face image captured using each of the different settings, and then averaged to calculate the mean volume for each image. This method enabled us to exclude any error that may occur from manual segmentation of B-scans, and, therefore, attribute any error from the calculated volumes to the change in imaging system settings.

### Measurement of Subretinal Bleb Volumes Based on MIOCT Images

With MIOCT, we imaged subretinal blebs formed by delivery of 50 μL diluted triamcinolone into the subretinal space of vitrectomized porcine eyes (purchased from an abattoir). Any leakage of diluted triamcinolone, which consisted of a suspension of white triamcinolone particles in clear BSS, was visible to the surgeon through the microscope and captured on MIOCT as reflective white particles.^28^ To perform subretinal injections, we used a 25 gauge/38 gauge MedOne PolyTip cannula (MedOne Surgical, Sarasota, FL) attached to a 1.0 mL syringe with flexible extension tubing. During the subretinal injection, we documented the surgeon's estimation of the percentage of leakage of triamcinolone from the retinotomy site or the cannula tip as well as any factors that may have caused any irregularities in the formation of the subretinal bleb. Each porcine eye was used only for one subretinal injection.

We imaged and recorded the injection and formation of the subretinal blebs using the swept-source MIOCT. We adjusted the MIOCT scan position based on the live 3D image to ensure the boundaries of the subretinal blebs were captured within the axial and lateral scan dimensions. Single volumetric images of the subretinal blebs at completion of the subretinal injection, immediately after the subretinal cannula was removed from the retinotomy, were used to analyze and calculate the volume delivered into the subretinal space. The MIOCT imaging system settings were the same as those used for the validation experiments described above.

**Figure 2:**
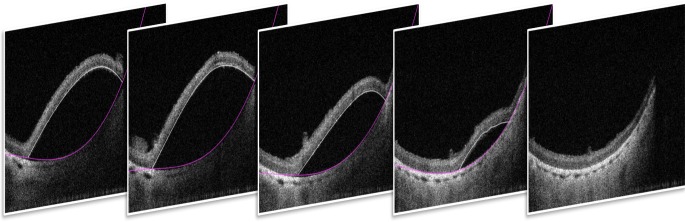
Selected samples of segmented B-scans from a single 3D volume of a subretinal bleb. In each B-scan, the area of occupied by the subretinal bleb, from the upper boundary immediately under the neurosensory retina to the lower boundary immediately above the retinal pigmented epithelium, is demarcated by the *white* and *purple lines*, respectively.

To calculate the volume using the acquired MIOCT volumetric images, we first manually segmented the area occupied by the subretinal bleb in each B-scan using DOCTRAP software.^25^ This involved tracing the upper boundary (lower margin of the neurosensory retina) and the lower boundary (upper border of the RPE; Fig. 2), from which we were able to obtain the total number of pixels occupied by the subretinal bleb. We multiplied the total segmented area by the calibrated voxel dimensions to calculate the volume of the subretinal bleb in microliters. We analyzed the subretinal bleb volumes in which the surgeon observed bleb formation with 50% or less leakage of triamcinolone, and excluded one volume with inadequate visualization of bleb margins.

To compare the accuracy of the MIOCT volume measurements to that of the current standard (volume injected as indicated by the delivery device with lack of surgeon-observed leakage), we calculated the image-based volumes of the subretinal blebs that were created without any surgeon-observed leakage of triamcinolone. We applied the image-based method to measure the volumes of subretinal blebs without any observed leakage. However, preliminary results showed a disagreement in volumes between the current standard and MIOCT image-based method, and, therefore, we proposed and tested the alternative hypothesis that MIOCT image-based volume measurements were more accurate than the surgeon's estimation of volume of triamcinolone leakage. We calculated the difference between the expected and measured volumes in all eyes.

To evaluate the reproducibility of MIOCT image-based volume measurements using different scan parameters, we imaged and analyzed a small subset of blebs using each of the following lateral scan dimensions and scan angles: 6 × 6 mm at 0°, 6 × 6 mm at 90°, 10 × 10 mm at 0°, and 10 × 10 mm at 90°. The bleb volume was calculated for each image acquired, and used to calculate the mean volume and standard deviation.

To assess intergrader reproducibility for manual segmentation of subretinal blebs, a second grader repeated the manual segmentation process described above for a random subset of blebs. The subretinal bleb volumes resulting from the manual segmentation by the second grader then were compared to those obtained by the first grader.

Additionally, to assess the accuracy and precision of the delivery device, we injected 50 μL distilled water onto a digital mass scale, and repeated the process 10 times each with 2 different syringes.

### Statistical Analysis

The accuracy of our MIOCT image-based volume measurement method was determined using propagation of uncertainty^26^ to calculate the overall uncertainty from the multiple steps in our method. Propagation of uncertainty combined the variances in measurement associated with the *X* and *Y* voxel dimensions and manual segmentation.

Reproducibility of image-based volumes acquired using different lateral scan dimensions, scan angles, and focal lengths was assessed using intraclass correlation and paired *t*-tests. Image-based volumes acquired using different focal length settings for the electrically-tunable lens were compared using a multivariate analysis of pair-wise differences among three focal lengths simultaneously based on Wilk's λ. Intergrader reproducibility of manual segmentation of subretinal blebs was assessed using a Wilcoxon signed rank test.

The image-based volumes of the subretinal blebs from injections without any surgeon-observed leakage were compared to the intended injection volume using absolute difference and percent error. For all subretinal blebs, the surgeon estimation of the amount of leakage was compared to the volume loss as measured using the MIOCT image-based method using a paired *t*-test to assess for significant difference and intraclass correlation to assess for agreement.

## Results

### Validation of MIOCT Image-Based Volume Measurements

The standard deviation in volume calculated from repeated manual segmentation (*n* = 5) of the ceramic ball of 5,968,131 voxels in the same volumetric image was 23,259 voxels, the standard deviation in the *X* voxel dimension was 0.009562 mm/pixel (*n* = 14 eyes), and the standard deviation in the *Y* voxel dimension was 0.06984 mm/pixel (*n* = 14 eyes). Using propagation of uncertainty calculations to combine the individual variances, we reported that our MIOCT image-based volume measurements are accurate to ±1.0 μL (6.0% of measured volume).

MIOCT image-based volume measurements were reproducible when imaging under different imaging system settings: lateral dimension, scan angle, and focal length (Table 1). There was no significant difference in image-based volume measurements of the ceramic ball when imaging the same ball in the same porcine eye with different lateral scan dimensions (*n* = 5 eyes, *P* = 0.73, paired *t*-test; Table 1) and intraclass correlation was 0.92 (95% confidence interval [CI], 0.53–0.99). There also was no significant difference when imaging with two different MIOCT scan angles (*n* = 5 eyes, *P* = 0.41, paired *t*-test; Table 1), and intraclass correlation was 0.77 (95% CI, 0.02–0.97). Varying the focal length of the electrically-tunable lens ±1.8 mm from ideal focus produced no significant differences among the three groups (*n* = 7 eyes, *P* = 0.26, multivariate analysis of pair-wise differences simultaneously based on Wilk's lambda; Table 1), and overall intraclass correlation was 0.74 (95% CI, 0.46–0.96). The image grader noted that some of the +1.8 mm images were not as sharply defined and, therefore, more difficult to measure.

**Table 1:**
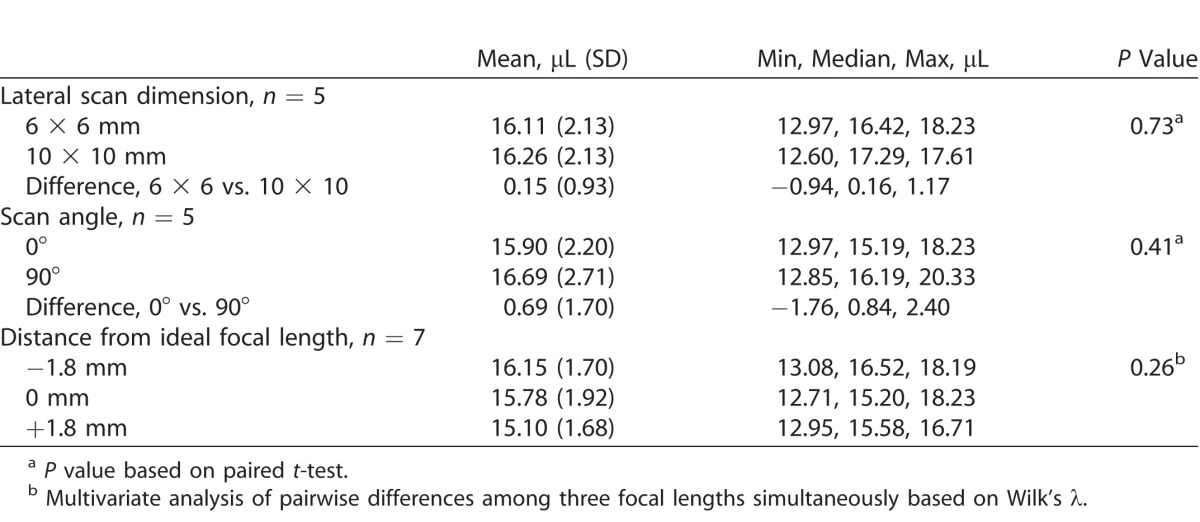
Reproducibility of MIOCT Image-Based Volume Measurements When Varying the Imaging System Settings by Using Different Lateral Dimensions, Scan Angles, and Focal Lengths on the Electrically-Tunable Lens While Maintaining Parfocality With the Surgical Microscope

### Measurement of Subretinal Bleb Volumes Based on MIOCT Images

**Figure 3:**
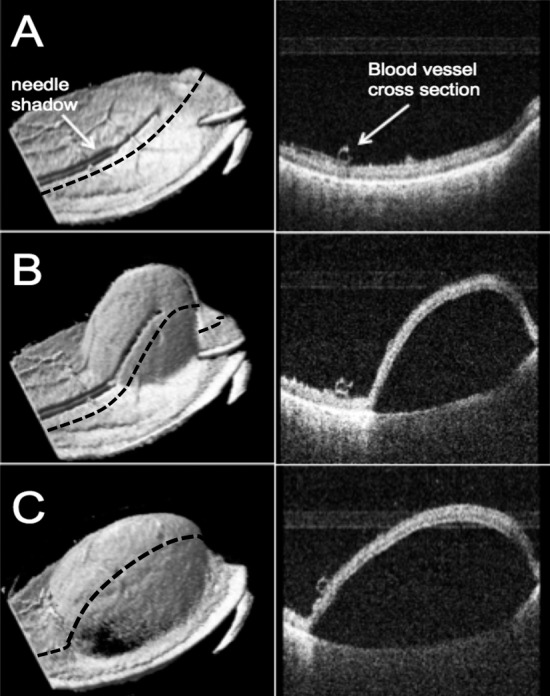
MIOCT 3D volumetric images (*left*) and a B-scan from the corresponding volume (*right*) of a subretinal injection (A) before, (B) during, and (C) at the end of the injection into the subretinal space. The position of the cross-sectional view provided by the B-scan was constant in the three images shown above, shown by the position of the *black dashed line*.

**Figure 4:**
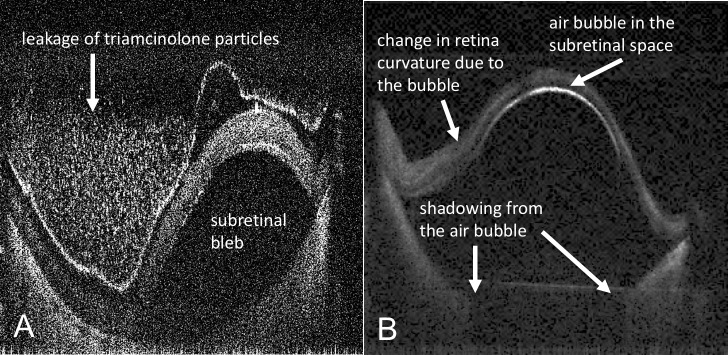
MIOCT B-scan images of (A) a subretinal bleb with triamcinolone leakage into the vitreous cavity and (B) a subretinal bleb with a volume of 30 μL, of which 7.3 μL was occupied by an air bubble that was not recognized by the surgeon through the microscope. The triamcinolone particles inside the subretinal bleb are not visible due to shadowing from the retina.

We obtained the MIOCT image-based volumes of 11 of 12 subretinal blebs, and excluded one bleb due to inadequate visualization of bleb margins. Swept-source MIOCT allowed for live 3D visualization of bleb formation as 50 μL of diluted triamcinolone was injected into the subretinal space (Fig. 3), as well as dynamic adjustment of the MIOCT scan location to capture all boundaries of the subretinal blebs. Triamcinolone leakage was visualized on MIOCT images (Fig. 4A).

The MIOCT image-based volumes of all 11 subretinal blebs were less than the target injection volume of 50 μL (mean MIOCT image-based volume: 19.8 ± 13.8 μL; mean difference from expected volume: 30.2 μL). In four of the 11 blebs, the surgeon did not observe any leakage of triamcinolone and, therefore, we expected all 50 μL to be delivered into the subretinal space. In contrast to the expected 50 μL, however, the four no-leakage blebs yielded a mean volume of 32 ± 12.5 μL. This is a mean absolute difference of 18 μL from expected, and mean percent error of 36 ± 25% (Table 2).

**Table 2:**
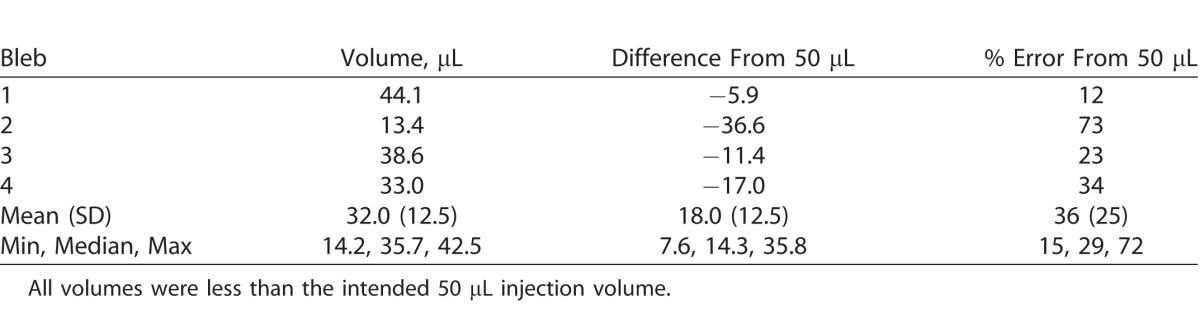
Image-Based Volume Measurements of Subretinal Blebs With No Surgeon-Observed Leakage

**Figure 5:**
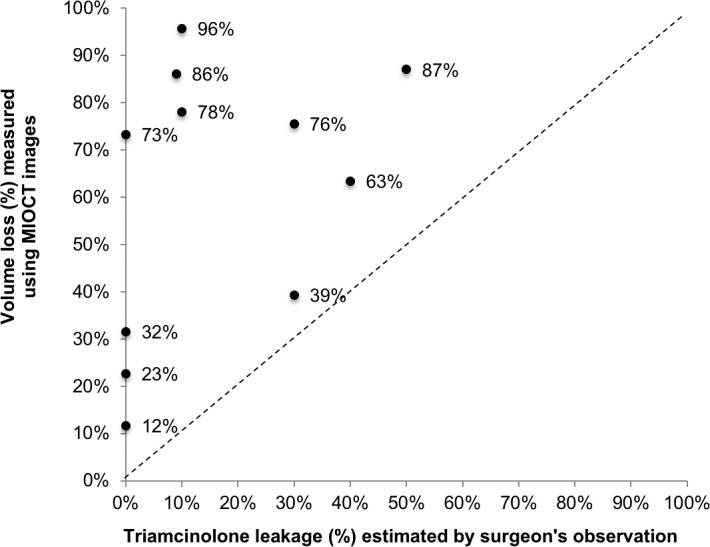
The percentage of subretinal injection volume loss as estimated by the surgeon compared to MIOCT image-based volume measurements (*n* = 11 subretinal injections). There is no correlation between the surgeon's estimations and the MIOCT image-based volumes. The percentages of volume loss as measured using MIOCT were all greater than those estimated by the surgeon.

For all 11 subretinal blebs, the surgeon's estimations of the percentage of leakage for each subretinal bleb was significantly different from and showed no correlation with the volume loss based on MIOCT image-based volume measurements (*P* < 0.001, *n* = 11, paired *t*-test; intraclass correlation, 0; 95% CI, 0–0.37; Fig. 5). Observations made by the surgeon during the subretinal injections included that leakages of triamcinolone occurred from the subretinal cannula before injection, from the retinotomy site during and after injection, and from the subretinal cannula after withdrawal from the retinotomy, and all were included in the surgeon's total estimation of leakage.

After generation of our alternative hypothesis, we also compared the surgeon's estimation of volume loss from the subretinal blebs to that measured using MIOCT. The MIOCT image-based volumes of all 11 subretinal blebs were less than the target injection volume of 50 μL (mean MIOCT image-based volume 19.8 ± 13.8 μL). Triamcinolone leakage was visualized on MIOCT images (Fig. 4A). Note that for one bleb, while the volume of the total bleb was 30 μL, 7.3 μL of this was occupied by an air bubble that was not recognized by the surgeon (Fig. 4B); the surgeon noted a sharply demarcated hyperreflective line at the inferior border of the detached retina, which upon review of MIOCT images then was understood to be the interface between the retina and the subretinal air bubble. For this tall bleb with the air bubble, the axial distance between the highest point on the retina and the lowest point on the upper border of RPE was approximately 2.7 mm.

We then evaluated the reproducibility of MIOCT image-based volume measurements using different scan parameters on a subset of blebs with an intended dose of 50 μL and no surgeon-observed leakage (*n* = 4, mean measured volumes range, 14.2–42.4 μL). Using the methods described above and changing the lateral scan dimension and scan angle, the standard deviation in measurements ranged from 0.5 to 2 μL (Table 3). We also evaluated intergrader reproducibility of manual segmentation using a random subset of subretinal blebs, and found no significant difference between bleb volumes segmented by two independent graders (*n* = 5, mean difference 1.0 ± 0.43 μL, *P* = 0.06, Wilcoxon signed rank test).

**Table 3:**
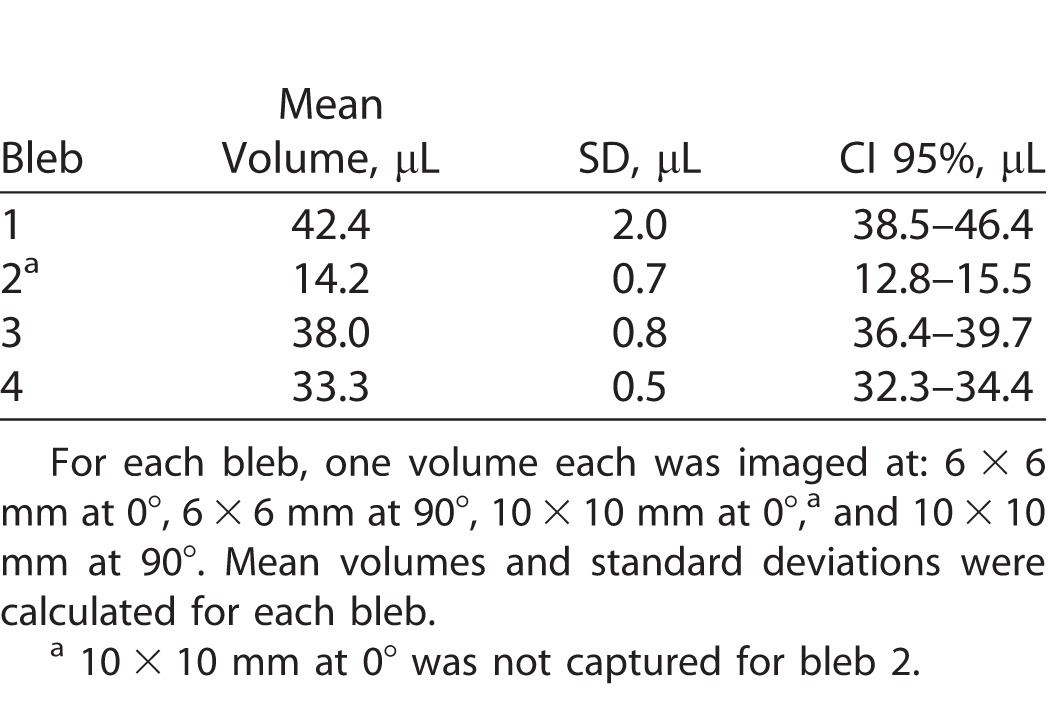
Evaluation of Reproducibility of MIOCT Image-Based Bleb Volume Measurements With Change in Scan Parameters of Lateral Scan Dimension and Scan Angle, Using a Subset of Blebs

Additionally, to ensure that the discrepancy between target volume and the volume delivered was not due to error in the delivery system (a 25 gauge/38 gauge MedOne PolyTip cannula attached to a 1.0 mL syringe with flexible extension tubing), an assessment of the accuracy and precision of the delivery system using a digital mass scale showed a mean measured volume of 49.2 ± 4.7 μL (*n* = 20, percent error, −2%; coefficient of variation, 10%).

## Discussion

To address the need for direct measurement of subretinal bleb volumes during surgery, we developed a MIOCT image-based method for volume measurement. Our validation results using a ceramic ball showed that use of MIOCT images allowed for accuracy of measurement of volumes within 6.0% of the actual volume, and were reproducible under different imaging system settings and in different porcine eyes. When we evaluated the reproducibility of our MIOCT image-based method to measure subretinal bleb volumes ranging from 14 to 43 μL, the standard deviation was small (0.5–2 μL). We then compared subretinal bleb volumes obtained from our validated MIOCT image-based method to the surgeon's estimation and the recorded volume injected from the calibrated delivery device. This revealed a much smaller actual bleb volume than reported from the calibrated delivery device or predicted from the surgeon estimate of volume loss.

Because we validated the accuracy and reproducibility of the MIOCT image-based method, the potential causes for the discrepancy between the intended volume injected from the syringe and the image-based volume of the subretinal bleb include reflux from the retinotomy, reflux into the syringe, or leakage from the cannula into the vitreous. It is possible that the diluted triamcinolone that was injected may have uneven distribution of the serous (BSS) and solid (triamcinolone particles) components, leading to reflux of the clear serous component that could not be visualized by the surgeon. These potential causes of discrepancies between the volume injected as observed from the syringe compared to the volume directly measured of the subretinal bleb are likely to be applicable to other preclinical and clinical studies of subretinal injection therapies. The results of this work support further development of such measurement methods for use in human eyes, particularly measurement of subretinal bleb volumes in patients receiving subretinal injections in clinical trials.

Previous studies also have demonstrated the use of OCT imaging in visualizing the delivery of subretinal fluid.^15,17,19^ These studies, which used SD-OCT, discussed limitations that hindered the successful imaging of the area of interest, including significant time required for close interaction between the operator and surgeon to localize the instrument to align and capture the SD-OCT images, and the limited depth of capture on B-scans.^15^ Accurate quantification of subretinal volumes requires imaging of all boundaries of the whole subretinal bleb. The use of swept-source MIOCT ^21,22^ addresses some of the limitations described previously in SD-OCT systems by enabling fast and dynamic localization of instruments and subretinal bleb boundaries as the bleb evolves, and also images depths up to 7.4 mm, more than the 2.5 mm depth offered by current generation posterior segment SD-OCT systems. One of the blebs in this report was 2.7 mm tall and would not have been imaged completely using an SD-OCT system. Additionally, increased imaging depth is useful particularly when blebs unexpectedly form more peripherally where the retina is curving upwards, increasing the axial height of the bleb. While SD-OCT can be used to visualize and possibly quantify subretinal bleb volumes, swept-source OCT does offer advantages that may benefit in future translation to in vivo imaging.

The limitations of this study include small sample size and not using a strictly standardized ratio of BSS to dilute the triamcinolone. Additionally, it would have been helpful to record not just the surgeon's estimation of the amount of leakage, but also the surgeon's estimated bleb size. Furthermore, manual segmentation of the volumetric OCT images is time consuming and could be automated.^29^

To translate MIOCT image-based volume measurements into human care, it would be appropriate to repeat these experiments in human eyes and automate the segmentation of the subretinal blebs. Rapid, real-time analysis of the amount of volume delivered successfully into the subretinal space, concurrent with the 3D visualization of the subretinal blebs forming in real time, has the potential to allow for real-time feedback on the volume of therapeutics delivered into the subretinal space. Although in our study we did not yet demonstrate the quantification of the subretinal bleb volumes in real time, we did clearly demonstrate the feasibility, accuracy, and reproducibility of this volume quantification technique using swept-source MIOCT imaging, as well as the importance of and urgent need for quantification and confirmation of the accuracy of doses of subretinal therapeutics delivered. Enhanced visualization and image-based volume measurements using MIOCT would allow for the surgeon to engage in real-time surgical decision-making to improve outcomes.

In conclusion, the volume of subretinally delivered therapeutics can be measured directly and reproducibly using MIOCT. Application of our image-based method to measure subretinal bleb volumes revealed that the intended volume might not be delivered into the subretinal space. Use of this technique will contribute to the accurate assessment of subretinal dose delivered, which is important in studies of efficacy and toxicity of retinal therapies.

## Summary

Use of MIOCT to measure subretinal injection volumes can allow for accurate and reproducible assessment of subretinal dose delivered, which may have therapeutic implications in studies evaluating the efficacy and toxicity of subretinal therapies.
